# The relationship between night work and involuntary weight change: data from the fifth Korea National Health and Nutrition Examination Survey (KNHANES 2010–2012)

**DOI:** 10.1186/s40557-016-0088-1

**Published:** 2016-01-29

**Authors:** Jongho Kwon, Jung-Woo Park, Jin-Soo Park, Seyoung Kim, Hyunrim Choi, Sinye Lim

**Affiliations:** Department of Occupational and Environmental Medicine, Kyung Hee University Hospital, Seoul, South Korea

**Keywords:** Night work, Weight change, KNHANES

## Abstract

**Background:**

Previous studies on the association between shift or night work and weight change have been focused on finding the risk of weight gain caused by shift or night work. In this study, we aimed to reveal the risk of weight gain and weight loss associated with night work by using a nationwide representative data.

**Methods:**

This study was performed on 1605 full-time wage workers between the age of 20 and 69 based on the fifth Korea National Health and Nutrition Examination Survey (KNHANES 2010–2012). The association between night work and involuntary weight change in the previous year was divided into the categories of weight gain and weight loss and studied with modifications in socio-demographic variables, health behavior-related variables, and occupational characteristic variables.

**Results:**

The participants working in night work accounted for 10.6 % of total study participants (male; 11.9 %, female 7.4 %). Workers who worked more than 48 hours per week on average accounted for 41.6 % of the total study participants (male; 46.3 %, female 29.1 %).

Odds ratio (OR) of weight loss associated with night work in male workers was 0.34 (95 % confidence interval (CI) 0.16–0.76) after controlling for several related factors. OR of weight loss associated with night work in female workers with long working hours was 1.95 (95 % CI 0.47–80.86) and that of weight gain was 2.83 (95 % CI 0.12–69.83) after adjusting associated factors.

**Conclusions:**

In this cross-sectional study with national representative sample, night work may lower the risk of weight loss in male workers and induce weight change (weight loss or weight gain) in female workers with long working hours. Therefore, future studies with cohort study design for night workers are needed to reveal the mechanisms and health effects of weight change associated with night work and establish proper management solutions with health and labor policies for Korean night workers.

## Background

It has been proposed that adjustments in working time are necessary with human activity hours expanding to 24 hours and the society becoming complex with the development of technology. The form of shift work using time other than normal day work hours based on economic rationality has come to the fore with shift work carried out in many industry fields as a method to improve productivity and to secure business competitiveness [1]. Although a clear definition for shift work has not been defined, all types of work outside the day work hours of 6 a.m. to 6 p.m. are considered as shift work which in many cases include night work. International Labour Organization (ILO) defines night work as “all work which is performed during a period of not less than seven consecutive hours, including the interval from midnight to 5 a.m.” [2].

According to the fifth European Working Conditions Survey (EWCS), 19 % of workers in 27 countries are engaged in shift work including night work, while in the U.S., around 15 % of full-time workers are engaged in shift work [3, 4]. In South Korea, the survey carried out by the Ministry of Employment and Labor in 2011 showed that 15.2 % of 3414 companies with 10 or more workers are engaged in shift work. According to the 2010 Korean Working Conditions Survey (KWCS), a high percentage of shift work was found in assembly of machinery and machine operation workers (24.3 %) and service workers (13.6 %) [5, 6].

Shift work is known to disturb the normal circadian rhythm causing sleep disorders and sleepiness during everyday life and work [7]. Such sleep disorders and sleepiness lower productivity during work and increase incidence of accidents [8], heighten job stress and depressed mood [9, 10], increase smoking and alcohol consumption, result in lack of exercise, and bring about changes in eating habits [11, 12]. In addition to these, it has been reported that disturbance of autonomic nervous system, inflammatory responses, and lipid and glucose metabolism resulting from shift work increased the incidence of cardiovascular and gastrointestinal diseases [13, 14]. There is also a limited evidence that shift work increased breast cancer incidence rates in female shift workers [15] and it has also been reported that it increases the risks of spontaneous abortion, low birth weight, and preterm birth [16]. Crucially, the effect of shift work is more pronounced in shift work including night work [17].

Modest weight change can be considered as a process of maintaining homeostasis, however, imbalance of weight like continuous weight gain or weight loss can cause obesity or underweight. In addition, weight changes are known to work as an independent factor in increasing the mortality [18, 19]. Weight gain is a risk factor of coronary heart disease, type 2 diabetes, and breast cancer [20–22]. In the case of weight loss, it can lead a decline in load of weight-bearing skeleton and it may cause changes in bone remodeling leading to bone loss. As a result, weight loss is a risk factor of hip fracture. Furthermore, weight loss can be an indicator of malnutrition or poor health state and it was significantly associated with poor self-reported health [23, 24]. Therefore, the study on weight change associated with occupational factors is being performed in various viewpoints [25, 26].

Previous studies on weight change associated with shift or night work have been mostly performed to examine the risk of weight gain caused by shift or night work. However, such relationship failed to show consistent results due to limitations of cross-sectional study design and insufficient adjusting for confounders [27]. Therefore, the possibility of weight loss from shift or night work has also been raised [28]. To examine the association between shift or night work and weight change accurately, it is needed to evaluate the effect of weight change by dividing into weight gain and weight loss. In addition, to exclude the positive health effect of voluntary weight change, it is necessary to explore only involuntary weight change. And a clear definition for shift work has not been defined, it is necessary to evaluate limited night work group that work a substantial number of hours in night (from midnight to 5 a.m). In this study, we aimed to explore the possibility of involuntary weight gain or weight loss associated with night work separately by applying the definition of night work by ILO with using a nationwide representative data.

## Methods

### Study subjects

The KNHANES is a survey used as a basis in calculating the statistical data needed in determining the status of health and nutrition of Koreans nationwide and in health policy establishment and evaluation. This survey was carried out from 1998 and conducted up to the fifth survey (2010–2012) with the sixth survey currently in progress. In the fifth KNHANES (2010–2012), the number of survey participants in each year was 8958 (participation rate; 81.9 %), 8518 (participation rate; 80.4 %), and 8058 (participation rate ; 80.0 %), respectively. The fifth KNHANES applied Rolling Sampling Survey method, therefore, the study participants became a probability sample representing Koreans nationwide. Sampling frameworks have been divided into ordinary residential and apartment areas with the residential registration data used for ordinary residential areas and the apartment market price trends used for apartment areas. Accordingly, 576 areas were selected with 20 households chosen per area through table of random numbers, amounting to 11,400 households. Interviews for questionnaire survey, nutritional examinations, and medical examinations were carried out on the 25,534 members of the selected households to produce total of around 600 types of socio-economic, occupational, nutritional, and health status information.

Among the fifth KNHANES total survey participants of 25,534 people, we restricted the study population of this study to full-time wage workers between the age of 20 and 69 and excluded voluntary weight changers, soldiers, pregnant women, and short-time workers working less than 10 hours per week. Finally, 1605 people were selected as the final analysis group.

### General characteristics

The gender-stratified analysis was performed through classification of male and female. Age was classified into units of 10 years between the age of 20 and 69 and the education level classified into less than high school, graduated from high school, and graduation from college or university. The socioeconomic status was classified into four levels by using the obtained data in household income quartiles, while the marital status was classified into single, married, and others (separated, divorced, and widow/widower).

Smoking was classified into current smoker for participants currently smoking and have smoked 100 cigarettes or more in the entire life, while the rest of the participants were classified as none or ex-smoker. For the alcohol consumption condition, men drinking seven glasses (five glasses for women) per occasion on average of once or more a week were classified as moderate or heavy alcohol consumption group, while those not applicable were classified as none or social. Exercise status was classified into light, moderate, and vigorous exercise according to the intensity and frequency of exercise. Moderate exercise was defined as five times or more a week and more than 30 minutes of moderate intensity of exercise (e.g., swimming, volleyball). Vigorous exercise was defined as three times or more a week and more than 20 minutes of vigorous intensity of exercise (e.g., soccer, basketball). The other was defined as light exercise.

### Occupational characteristics

The labor status was classified into non-manual work for government officials, managers, professionals, and office workers, while those working in services, sales and agriculture and fishery industries, and factory workers were classified as manual work. Working more than 48 hours per week were defined as long working hours by ILO standard [29], therefore, the work hours per week was classified into 48 hours or less group and more than 48 hours group.

### Night work

In KNHANES, shift work group was decided by the question, “Do you work mostly during the day time (between 6 a.m. ~ 6 p.m.)? Or do you work at a different time period?”. Participants who answered “Mostly at day time” were classified as day workers, while the rest of the participants who answered “Evening shift (between 2 p.m. ~ midnight), night shift (between 9 p.m. ~ 8 a.m. next day), regular day and night rotating shift, 24-hour rotating shift, split shift (working two shifts in one day), irregular rotating shift, and others” were classified as shift workers. In this study, evening shift group, split shift group, and others which are unlikely to meet the definition of night work have been eliminated from the final analysis to explore the health effects associated with shift work including night work (working over 7 consecutive hours including the interval from midnight and 5 a.m.) defined by the ILO [2]. In the final analysis, the day work group was decided as the control group, while participants who answered night shift, regular day and night rotating shift, 24-hour rotating shift, and irregular shift were decided as the night work group. In the case of irregular shift, there is no more intensive information about irregular shift system in KNHANES. However, most train drivers and controllers with irregular shift systems participating in one study in Finland included night work in their shift schedules, therefore, we included irregular shift in the night work group [30].

### Involuntary weight change

Weight change is considered clinically significant if there is a loss of over 5 % of one’s weight within 6 months to a year [31], whereas weight gain of more than 2 kg in a year has been suggested as a significant weight gain, although there is no clinical consensus on weight gain other than Body Mass Index (BMI) standard for obesity [32]. We used two questions in KNHANES to evaluate weight change and voluntariness; “Were there any changes in weight compared to the previous year?” and “Have you ever tried to control weight voluntarily in the previous year?”. For the question about weight change, the answer was categorized with “No changes (Weight changes of less than 3 kg)”, “Lost weight”, and “Gained weight” and for the question about voluntariness, the answer was classified as “Tried to lose weight”, “Tried to maintain current weight”, “Tried to gain weight”, and “Never tried to control weight”.

In this study, we excluded the subjects that answered with “Tried to maintain current weight”, “Tried to lose weight”, and “Tried to gain weight”. The reasons are that these subjects voluntarily changed or maintained their weight. As the final analysis subjects, among the subjects that answered “Never tried to control weight”, the subjects that answered with “No changes (weight changes of less than 3 kg)” were classified as the no weight change group, while the subjects that answered with “Lost weight” as the weight loss group, and “Gained weight” as the weight gain group.

### Statistical analysis

The fifth KNHANES applied complex sampling design to improve the sample’s representativeness and the accuracy of the estimation. To estimate unbiased outcomes in the average, prevalence rate, and risk ratio, stratified variables, cluster variables, and weights should be applied. Weight used in fifth KNHANES has two levels to represent total households and population of South Korea; household weight for household level analysis and individual weight for individual level analysis. Final weights were created through several steps, such as, calculating the base design weight, adjusting for non-response, post-stratification adjustment, and extreme value trimming. This study used complex sample module of SPSS. The module was operated to estimate the frequency of a population from the sampling data by using stratified variables, cluster variables, and weights. After applying statistical algorithms in the module, total subjects of this study became 4,077,302.

To figure out the general characteristics of the study participants, the frequency and the configuration fraction of each variable were presented through the stratification of total participants, male, and female. Then chi-square tests were conducted on weight loss and weight gain, respectively, and compared to the no weight change group to analyze the relationship between several relating factors associated with weight loss and weight gain. Odds ratio (OR) was calculated by using logistic regression analysis to determine the association between night work and involuntary weight change. It was constructed into a three-level model, with the first model using unadjusted logistic regression analysis and the second model using multivariate logistic regression analysis with adjusted socio-demographic factors (age, education, household income, and marital status). Socio-demographic factors, health behavior-related factors (smoking, alcohol drinking, and physical activity), and occupational factors (type of job and working hours per week) were all adjusted in the final model. Then, the average working hours per week have been stratified by 48 hours or less group and more than 48 hours group and logistic regression analysis conducted to explore OR influenced by long working hours. SPSS version 19.0 was used for the statistical analysis and the statistical significance level was set at *p* < 0.05.

### Ethics statement

This study was performed by using the open data of the fifth KNHANES. All study participants of the fifth KNHANES signed a consent form agreeing to the purposes of the survey and this survey has been approved by the Institutional Review Board (IRB) of the Korea Centers for Disease Control and Prevention (KCDC).

## Results

### General characteristics of the study participants

Among the total study participants, male participants accounted for 72.6 % and female 27.4 %. In terms of occupational characteristics, the percentage of manual work for male was slightly higher than that of female. Workers who worked more than 48 hours per week on average as long working hours accounted for 41.6 % of the total study participants, and the percentage of long working hours in male was 46.3 % and significantly higher than that of female (29.1 %) (Table 1).

**Table 1:** General characteristics of the study subjects

	Total		Male		Female	
Variable	n^a^	n^b^(%^c^)	n^a^	n^b^(%^c^)	n^a^	n^b^(%^c^)
Total	1605	4077302(100.0)	1075	2960342(100.0)	530	1116959(100.0)
Age(years)						
20–29	216	821331(20.1)	116	529449(17.9)	100	291882(26.1)
30–39	474	1230449(30.2)	343	980278(33.1)	131	250171(22.4)
40–49	401	1068241(26.2)	285	794654(26.8)	116	273587(24.5)
50–59	315	654930(16.1)	205	458585(15.5)	110	196345(17.6)
60–69	199	302351(7.4)	126	197377(6.7)	73	104974(9.4)
Education						
≤ Middle school	347	782262(19.2)	192	493489(16.7)	155	288773(25.9)
High school	540	1500737(36.8)	366	1120342(37.8)	174	380394(34.1)
≥ University	718	1794303(44.0)	517	1346511(45.5)	201	447792(40.1)
Income						
1st quartile	137	328103(8.0)	74	196406(6.6)	63	131697(11.8)
2nd quartile	443	1204464(29.5)	313	927339(31.3)	130	277125(24.8)
3rd quartile	552	1445150(35.4)	371	1043236(35.2)	181	401914(36.0)
4th quartile	473	1099584(27.0)	317	793362(26.8)	156	306223(27.4)
Marital status						
Single	262	934340(22.9)	154	630167(21.3)	108	304173(27.2)
Married	1252	2931100(71.9)	893	2243410(75.8)	359	687690(61.6)
Others	91	211862(5.2)	28	86766(2.9)	63	125096(11.2)
Alcohol drinking						
None or social	1159	2837667(69.6)	674	1832693(61.9)	485	1004975(90.0)
Moderate or heavy	446	1239634(30.4)	401	1127650(38.1)	45	111985(10.0)
Smoking						
None or ex-smoker	999	2322062(57.0)	503	1289687(43.6)	496	1032375(92.4)
Current smoker	606	1755240(43.0)	572	1670656(56.4)	34	84585(7.6)
Physical activity						
Light	1383	3462054(84.9)	918	2484421(83.9)	465	977633(87.5)
Moderate	91	242915(6.0)	52	155782(5.3)	39	87133(7.8)
Vigorous	131	372332(9.1)	105	320139(10.8)	26	52193(4.7)
Type of job						
Non-manual	713	1771262(43.4)	479	1257519(42.5)	234	513743(46.0)
Manual	892	2306040(56.6)	596	1702823(57.5)	296	603217(54.0)
Working hours per week						
≤ 48	957	2380084(58.4)	570	1588447(53.7)	387	791637(70.9)
> 48	648	1697218(41.6)	505	1371896(46.3)	143	325322(29.1)
Work schedule						
Day work	1418	3643616(89.4)	923	2609320(88.1)	495	1034295(92.6)
Night work	187	433686(10.6)	152	351022(11.9)	35	82664(7.4)
Night shift	28	74393(17.2)	18	48319(13.8)	10	26074(31.5)
Regular shift	78	201489(46.5)	62	164707(46.9)	16	36781(44.5)
24 hour shift	51	92821(21.4)	46	84865(24.2)	5	7956(9.6)
Irregular shift	30	64983(15.0)	26	53130(15.1)	4	11853(14.3)
Weight change						
No weight change	1248	3042783(74.6)	847	2219990(75.0)	401	822794(73.7)
Weight loss	142	381040(9.3)	92	278769(9.4)	50	102270(9.2)
Weight gain	215	653478(16.0)	136	461583(15.6)	79	191895(17.2)

^a^unweighted count

^b^estimated population size

^c^column and estimated percentage

The participants working in night work accounted for 10.6 % of total study participants with the percentage of night work in male (11.9 %) higher than female (7.4 %). The percentage of night work by shift type was 46.5 % for regular shift work, 21.4 % for 24-hour shift work, 17.2 % for night shift work, and 15.0 % for irregular shift work, in order. In terms of distinguishing differences between male and female participants, female showed a lower percentage in the 24-hour shift work compared to male (male: 24.2 %, female: 9.6 %) and a higher percentage in night shift work (male: 13.8 %, female: 31.5 %) (Table 1).

The male participants working in night work was significant in the age group of 60s, low level of educational attainment, manual workers, and working more than 48 hours a week. The female participants working in night work was significant in moderate or heavy alcohol consumption group (Table 2).

**Table 2:** General characteristics of male and female by work schedule

	Male		Female	
Variable	Day work	Night work	Day work	Night work
	n^a^(%^b^)	n^a^(%^b^)	n^a^(%^b^)	n^a^(%^b^)
Total	2609320(100.0)	351022(100.0)	1034295(100.0)	82664(100.0)
Age(years)^c^				
20–29	468087(17.9)	61363(17.5)	261607(25.3)	30275(36.6)
30–39	875124(33.5)	105154(30.0)	232659(22.5)	17512(21.2)
40–49	738135(28.3)	56519(16.1)	253412(24.5)	20175(24.4)
50–59	404736(15.5)	53848(15.3)	185802(18.0)	10543(12.8)
60–69	123239(4.7)	74138(21.1)	100814(9.7)	4160(5.0)
Education^c^				
≤ Middle school	422070(16.2)	71419(20.3)	277763(26.9)	11010(13.3)
High school	945666(36.2)	174676(49.8)	342680(33.1)	37714(45.6)
≥ University	1241584(47.6)	104927(29.9)	413852(40.0)	33940(41.1)
Income				
1st quartile	178888(6.9)	17518(5.0)	128559(12.4)	3138(3.8)
2nd quartile	835447(32.0)	91892(26.2)	253419(24.5)	23706(28.7)
3rd quartile	902113(34.6)	141123(40.2)	370488(35.8)	31426(38.0)
4th quartile	692873(26.6)	100489(28.6)	281829(27.2)	24394(29.5)
Marital status				
Single	538421(20.6)	91746(26.1)	274416(26.5)	29757(36.0)
Married	1988519(76.2)	254890(72.6)	645458(62.4)	42232(51.1)
Others	82380(3.2)	4386(1.2)	114422(11.1)	10674(12.9)
Alcohol drinking^d^				
None or social	1592309(61.0)	240384(68.5)	944300(91.3)	60675(73.4)
Moderate or heavy	1017012(39.0)	110638(31.5)	89995(8.7)	21989(26.6)
Smoking				
None or ex-smoker	1111880(42.6)	177806(50.7)	964964(93.3)	67411(81.5)
Current smoker	1497440(57.4)	173216(49.3)	69331(6.7)	15253(18.5)
Physical activity				
Light	2174814(83.3)	309607(88.2)	910681(88.0)	66952(81.0)
Moderate	147376(5.6)	8406(2.4)	82729(8.0)	4405(5.3)
Vigorous	287130(11.0)	33010(9.4)	40886(4.0)	11307(13.7)
Type of job^c^				
Non-manual	1212165(46.5)	45354(12.9)	487665(47.1)	26078(31.5)
Manual	1397155(53.5)	305668(87.1)	546630(52.9)	56586(68.5)
Working hours per week^c^				
≤ 48	1471445(56.4)	117002(33.3)	734250(71.0)	57386(69.4)
> 48	1137876(43.6)	234020(66.7)	300045(29.0)	25278(30.6)
Weight change				
No weight change	1938500(74.3)	281489(80.2)	764035(73.9)	58759(71.1)
Weight loss	259215(9.9)	19554(5.6)	96299(9.3)	5971(7.2)
Weight gain	411605(15.8)	49978(14.2)	173961(16.8)	17934(21.7)

^a^estimated population size

^b^column and estimated percentage

^c^significantly different between day work and night work in male subjects (*p* < 0.05)

^d^significantly different between day work and night work in female subjects (*p* < 0.05)

### Involuntary weight change and its association with relevant variables

In regards to involuntary weight change, weight loss in male participants was significant in the age group of 20s and 30s and manual workers. Weight gain in male was significant in the age group of 20s and 30s, high level of educational attainment, single, and moderate or heavy alcohol consumption group (Table 3).

**Table 3:** Involuntary weight change according to characteristics in male subjects

	No weight change	Weight loss	Weight gain
Variable	n^a^(%^b^)	n^a^(%^b^)	n^a^(%^b^)
Total	2219990(100.0)	278769(100.0)	461583(100.0)
Age(years)^c,d^			
20–29	309365(13.9)	61956(22.2)	158128(34.3)
30–39	638936(28.8)	129157(46.3)	212184(46.0)
40–49	687560(31.0)	48748(17.5)	58346(12.6)
50–59	416913(18.8)	15891(5.7)	25780(5.6)
60–69	167216(7.5)	23016(8.3)	7145(1.5)
Education^d^			
≤ Middle school	415384(18.7)	41724(15.0)	36381(7.9)
High school	809988(36.5)	121416(43.6)	188938(40.9)
≥ University	994617(44.8)	115629(41.5)	236265(51.2)
Income			
1st quartile	128890(5.8)	37409(13.4)	30107(6.5)
2nd quartile	690811(31.1)	81863(29.4)	154665(33.5)
3rd quartile	784316(35.3)	109728(39.4)	149192(32.3)
4th quartile	615973(27.7)	49770(17.9)	127619(27.6)
Marital status ^d^			
Single	406539(18.3)	80784(29.0)	142844(30.9)
Married	1749396(78.8)	188786(67.7)	305228(66.1)
Others	64055(2.9)	9199(3.3)	13512(2.9)
Alcohol drinking^d^			
None or social	1410436(63.5)	177538(63.7)	244719(53.0)
Moderate or heavy	809553(36.5)	101232(36.3)	216864(47.0)
Smoking			
None or ex-smoker	1022453(46.1)	96115(34.5)	171118(37.1)
Current smoker	1197536(53.9)	182654(65.5)	290465(62.9)
Physical activity			
Light	1848629(83.3)	219257(78.7)	416536(90.2)
Moderate	123131(5.5)	21311(7.6)	11341(2.5)
Vigorous	248230(11.2)	38202(13.7)	33707(7.3)
Type of job^c^			
Non-manual	965184(43.5)	74162(26.6)	218173(47.3)
Manual	1254805(56.5)	204607(73.4)	243411(52.7)
Working hours per week			
≤ 48	1183501(53.3)	119901(43.0)	285045(61.8)
> 48	1036489(46.7)	158868(57.0)	176538(38.2)
Work schedule			
Day work	1938500(87.3)	259215(93.0)	411605(89.2)
Night work	281489(12.7)	19554(7.0)	49978(10.8)

^a^estimated population size

^b^column and estimated percentage

^c^significantly different between no weight change and weight loss subjects (*p* < 0.05)

^d^significantly different between no weight change and weight gain subjects (*p* < 0.05)

Weight loss in female was significant in the age group of 30s. Weight gain in female participants was significant in the age group of 30s and 40s, low educational attainment, moderate or heavy alcohol consumption group, and current smokers (Table 4).

**Table 4:** Involuntary weight change according to characteristics in female subjects

	No weight change	Weight loss	Weight gain
Variable	n^a^(%^b^)	n^a^(%^b^)	n^a^(%^b^)
Total	822794(100.0)	102270(100.0)	191895(100.0)
Age(years)^c,d^			
20–29	216836(26.4)	25752(25.2)	49294(25.7)
30–39	148273(18.0)	39560(38.7)	62338(32.5)
40–49	191374(23.3)	18203(17.8)	64009(33.4)
50–59	174512(21.2)	10948(10.7)	10886(5.7)
60–69	91799(11.2)	7807(7.6)	5368(2.8)
Education^d^			
≤ Middle school	245145(29.8)	23029(22.5)	20599(10.7)
High school	241590(29.4)	31565(30.9)	107239(55.9)
≥ University	336059(40.8)	47676(46.6)	64057(33.4)
Income			
1st quartile	96039(11.7)	13273(13.0)	22386(11.7)
2nd quartile	202988(24.7)	30086(29.4)	44051(23.0)
3rd quartile	299364(36.4)	32570(31.8)	69980(36.5)
4th quartile	224403(27.3)	26341(25.8)	55478(28.9)
Marital status			
Single	235649(28.6)	19664(19.2)	48860(25.5)
Married	489691(59.5)	69149(67.6)	128851(67.1)
Others	97454(11.8)	13458(13.2)	14184(7.4)
Alcohol drinking^d^			
None or social	766987(93.2)	93006(90.9)	144982(75.6)
Moderate or heavy	55807(6.8)	9264(9.1)	46913(24.4)
Smoking^d^			
None or ex-smoker	775650(94.3)	94836(92.7)	161889(84.4)
Current smoker	47144(5.7)	7435(7.3)	30006(15.6)
Physical activity			
Light	714667(86.9)	81249(79.4)	181717(94.7)
Moderate	65375(7.9)	16373(16.0)	5385(2.8)
Vigorous	42752(5.2)	4649(4.5)	4793(2.5)
Type of job			
Non-manual	384975(46.8)	44607(43.6)	84161(43.9)
Manual	437819(53.2)	57663(56.4)	107734(56.1)
Working hours per week			
≤ 48	588048(71.5)	68562(67.0)	135026(70.4)
> 48	234745(28.5)	33708(33.0)	56869(29.6)
Work schedule			
Day work	764035(92.9)	96299(94.2)	173961(90.7)
Night work	58759(7.1)	5971(5.8)	17934(9.3)

^a^estimated population size

^b^column and estimated percentage

^c^significantly different between no weight change and weight loss subjects (*p* < 0.05)

^d^significantly different between no weight change and weight gain subjects (*p* < 0.05)

### The relationship between night work and involuntary weight change

To explore the relationship between weight change and night work, logistic regression analysis in conformity with the three-level model was carried out after stratification by gender. In addition to this, the average working hours per week were stratified by 48 hours or less group and more than 48 hours group to figure out OR influenced by long working hours and conducted logistic regression analysis (Table 5).

**Table 5:** Odds ratios (OR) and 95 % confidence intervals (CI) for involuntary weight change

		Male			Female		
		Model 1^a^	Model 2^b^	Model 3^c^	Model 1^a^	Model 2^b^	Model 3^c^
Weight loss							
	Day work	1.00	1.00	1.00	1.00	1.00	1.00
	Night work	0.52(0.24–1.11)	0.44(0.20–0.95)	0.34(0.16–0.76)	0.81(0.22–2.93)	0.66(0.15–2.84)	0.58(0.14–2.40)
Stratification by working hours per week							
≤48	Day work	1.00	1.00	1.00	1.00	1.00	1.00
	Night work	0.25(0.42–1.45)	0.23(0.36–1.50)	0.22(0.03–1.50)	0.60(0.12–3.05)	0.57(0.10–3.16)	0.47(0.08–2.93)
>48	Day work	1.00	1.00	1.00	1.00	1.00	1.00
	Night work	0.56(0.24–1.34)	0.44(0.16–1.20)	0.35(0.12–1.00)	1.64(0.17–16.07)	1.43(0.08–27.08)	1.95(0.47–80.86)
Weight gain							
	Day work	1.00	1.00	1.00	1.00	1.00	1.00
	Night work	0.84(0.44–1.59)	0.92(0.46–1.84)	0.98(0.48–2.02)	1.34(0.40–4.54)	1.19(0.33–4.30)	0.80(0.22–2.85)
Stratification by working hours per week							
≤48	Day work	1.00	1.00	1.00	1.00	1.00	1.00
	Night work	0.57(0.17–1.90)	0.53(0.15–1.93)	0.49(0.13–1.92)	0.57(0.12–2.76)	0.53(0.10–2.67)	0.34(0.40–2.87)
>48	Day work	1.00	1.00	1.00	1.00	1.00	1.00
	Night work	1.19(0.55–2.61)	1.75(0.70–4.28)	1.55(0.57–4.18)	5.04(0.77–32.89)	9.58(0.83–110.20)	2.83(0.12–69.83)

^a^Model 1: crude model

^b^Model 2: adjusted with socio-demographic factors (age, education, household income, and marital status)

^c^Model 3: adjusted with socio-demographic factors (age, education, household income, and marital status), health behavior-related factors (smoking, alcohol drinking, and physical activity), and occupational factors (type of job and working hours per week)

Weight loss in male workers associated with night work showed that the risk was significantly low in night work group (OR 0.34, 95 % confidence interval (CI) 0.16–0.76) after controlling for several related factors. However, OR for weight gain associated with night work was not significant in male workers. In the case of female workers, ORs for weight loss and weight gain associated with night work were not significant. However, the results after stratifying by average weekly working hours revealed that ORs were higher than 1.0 in more than 48 hours working group with 1.95 (95 % CI 0.47–80.86) in weight loss group and 2.83 (95 % CI 0.12–69.83) in weight gain group.

## Discussion

This study evaluated the risk of involuntary weight change associated with night work in Korean full-time wage workers from the nationwide representative data and revealed that night work may lower the risk of weight loss in male workers and induce weight change (loss or gain) in female workers with long working hours.

Most previous studies stated that there was a high risk of weight gain or obesity development resulting from shift or night work. Antunes [33] reviewed numerous articles related to metabolic syndrome, dyslipidemia, unhealthy eating habits, and increase in BMI and waist-hip ratio (WHR) associated with shift work and argued the risk of weight gain related with shift work [33]. Also, Drongelen [34] suggested a relationship between shift work and weight gain which was consistent to the statement presented earlier by Antunes [33] in a review article restricting with longitudinal studies on shift work exposed to night work between midnight and 6 a.m. [34]. Weight gain associated with shift work can be explained through the complex mechanisms brought on by the disturbance of circadian rhythm. First of all, changes in night and day patterns associated with shift work will reduce the production of the appetite-suppressing hormone, leptin, which is produced normally during the night while sleeping, and instead increase the production of the appetite enhancing hormone, ghrelin, resulting in stimulation of the appetite. As a result of this, the weight increases due to increased high calorie diet and late-night meals. Also, the disturbance of circadian rhythm leads to weight gain by increasing insulin resistance and bringing changes in the glucose and lipid homeostasis. In addition to this, the effects of reduction in physical activity during spare time, reduced social and family relationship, and job stress accelerate weight gain [33, 34].

Despite various evidences on weight gain associated with shift work suggested in the previous studies, weight loss associated with shift work has been reported in a one-year follow-up cohort study observing 377 new workers in the Netherlands [28]. In this Dutch study, Amelsvoort [28] assumed that the growth in smoking rate, the change in the neuro-endocrine secretion of hormones due to sleep problems, and the disturbance of the metabolic system increased the risk of weight loss. In addition to these interpretations, Amelsvoort [28] pointed out that the growth in smoking rate was not sufficient to explain the total effect, the disturbance of the metabolic system could induce weight loss only in short term, and BMI could increase after several years of shift work with increasing energy intake. Furthermore, Amelsvoort [28] assumed that the result was influenced by a behavioral change of the participants caused by the realization of the study itself.

On the contrary, some studies evaluated weight change with using the average value of BMI or by conducting long-term follow-up observations [35, 36]. However, such studies had the problem of not being able to determine the risk of weight gain and weight loss separately in shift workers, because the average value of BMI or the amount of weight change was used. For example, participants showing weight gain accounted for 42.9 % in the 14-year follow-up study of Suwazono [35] observing the changes in weight. This finding explained the reason why the impact of weight gain may be relatively significant when examining the risk of weight change with applying the average value of weight change. However, in the study of Kivimäki [25] for the association between job stress and weight change, the risk of weight gain and weight loss was separately evaluated. For five to eight years of follow-up, 29.2 % of male participants were weight loss and 70.7 % of them were weight gain. Among male participants, lower control group was significantly high risk in weight loss with bottom quintile in baseline BMI and higher strain group was significantly high risk in weight gain with top quintile in baseline BMI [25].

This cross-sectional study showed that night work may effect differently by gender on body weight. According to this study for male workers, night work may decrease the risk of weight loss with statistical significance compared with day work and increase the risk of weight gain with long working hours without statistical significance. In a previous study, Roos [37] reported that the risk of weight gain associated with shift work including night shift was elevated (OR 1.25, 95 % CI 0.81–1.82) in male workers and OR of weight gain associated with shift work with no night shift was similar. However, long working hours (>50 hours) may lower the risk of weight gain (OR 0.87, 95 % CI 0.41–1.83) without statistical significance in Finnish male workers. The results of Roos’ study were different from this study because some difference between Roos’ study and this study. The study design of Roos’ study was a prospective cohort study with a questionnaire survey and this study is a cross-sectional survey. Furthermore, industrial history of the country as well as working hours is quite different between Finland and Korea. Because of this different nature of the societies, the results might not be the same direction. Besides, the combined effect of both shift work and long working hours was not evaluated in Roos’ study, but this study. Long working hours as well as shift work is an established risk factor of workers’ health. Shift work with long working hours may more aggravate workers’ health compared with day workers without overtime work. In most previous studies, both factors were analyzed seperately. As shown in Liu’s study (2002), the proportion of shift workers was highest in the long working hour group (more than 60 hours per week) in Japan [38]. However, the combined health effects of both long working hours and shift work were not assessed in this study, even though the short sleeping time was included as a proxy of shift work. Korea is one of the longest working countries in OECD countries. Therefore, future studies are needed to elucidate the combined aggravating health effects of both long working and shift work in Korea.

On the contrary in the case of female workers, night work may induce weight change (weight loss or weight gain) with long working hours, even though the results were not statistically significant. In the aspect of weight gain, we suggest the possibility of weight gain caused by job stress as a medium. Kivimäki [25] already argued this by using the Whitehall II study and there can be bidirectional results for weight gain and weight loss associated with job stress [25]. In female participants, the higher follow-up BMI was slightly associated with higher job demand after adjusting with age, grade, and baseline BMI. Previous studies revealed that such job stress was more significant in shift work group than day work group, especially in a low level of job control [39, 40]. Therefore, there is a possibility of weight gain resulting from shift work in association with low job control. In Roos’ study (2013), the authors reported that the risk of weight gain associated with shift work including night shift was higher than that of male workers and showed a statistical significance. OR of weight gain associated with shift work with night shift was 1.37 (95 % CI 1.08–1.74) and the risk of weight gain associated with shift work with no night shift was decreased in female workers [37]. In addition, the risk of weight gain was slightly increased (OR 1.03, 95 % CI 0.64–1.65) in female workers with long working hours (>50 hours), even though the risk was statistically insignificant. Furthermore, female shift workers with long working hours have no time to rest, exercise, and have social relationships with their friends and families.

For explaining the possibility of the weight loss with night work, we suggest several hypotheses related to weight loss associated with night work in female workers with long working hours. First explanation is the possibility of weight loss caused by depressive symptoms and gastrointestinal disorders known to occur highly in shift workers. Driesen [9] reported that OR for depressive symptoms in shift workers was 2.05 in male (95 % CI 1.52–2.77) and 5.96 in female (95 % CI 2.83–12.56), compared to day workers. According to studies with Korean workers, Kim [41] reported a higher prevalence of irritable bowel syndrome and functional dyspepsia in female shift workers and Cho [42] reported depressive symptoms related with job stress and sleep quality in female workers. Knutsson [14] announced that shift workers showed increased incidence for gastrointestinal symptoms and peptic ulcer through the review about shift work and gastrointestinal disorders. Such a series of health effects related to shift work has been reported as a common cause of weight loss [31]. Therefore, depressive symptoms and gastrointestinal disorders caused by shift work may contribute to weight loss in shift workers. Second explanation is the suggestion in Kivimäki’s study (2006) for explaining the significant higher risk of weight loss in the low control group in bottom quintile in baseline BMI, even though the results were from male participants [25]. Kivimäki explained that it was influenced by the loss of appetite due to stress and suppression of upper gastrointestinal motility caused by activation of the sympathetic nervous system. In spite of these possibilities, the increased risk of weight loss related to night work was limited to long working female workers in our study without statistical significance. Furthermore, the risk of weight loss associated with night work was significantly low in male workers. Therefore, further studies are needed to explain these findings in this country.

We suggest several hypotheses to explain gender difference in the effect on weight associated with night work with long working hours. We present two factors related with the characteristics of Korean society. First factor is that women have experienced higher domestic labor burden than men. According to Korea’s Time Use Survey conducted in 2009, among double-income households, women used 185 minutes for housework, while men used 27 minutes. Therefore, Korean women disproportionately lack in time for sleep and recovery from work compared to Korean men [43], showing increased vulnerability to stress caused by long working hours. Second factor is that women are subject to discrimination in terms of promotion and wage and the risk of depressive symptoms related to organization injustice is higher for female than male [44]. According to previous studies, disturbances of circadian rhythm and sleep associated with shift work could cause weight gain. However, gender difference in weight gain associated with sleep disturbance was unclear [45]. In Korean society that have forced high domestic labor burden on women, sleep disturbance is more likely to occur in female workers with night work with long working hours and the effects have been reflected in our results.

This study has several limitations. Firstly, this study used data of the fifth KNHANES with a cross-sectional design to explore the current status of the population. Therefore, the participant’s weight change during the previous year was assessed instead of following of body weight of participants. Secondly, the participant’s period of time engaged in night work was not considered. In KNHANES, the only information available for shift work is the work schedule and work type and there is no data on duration of shift work. In the study of Amelsvoort [28], although weight loss was shown for one year follow up after starting shift work, there is also the possibility of weight gain after many years of shift work [28]. So the risk of weight change in night workers shown in this study may be a short term effect like in the study of Amelsvoort [28], especially about the risk of weight loss. Therefore, the risk of weight gain and weight loss should be figured out by considering the period engaged in night work in follow-up studies in the future. Thirdly, there is the possibility of information bias resulting from the use of participant’s self-report survey data on weight change during the previous year. However, we excluded participants with intentional weight change (loss or gain or maintenance) to overcome the limitation of a cross-sectional study design. In spite of these limitations, this study has secured the representativeness and credibility by using the KNHANES which determines the status of health and nutrition of Koreans nationwide. In addition, this study evaluated the risk of involuntary weight change associated with night work in Korean full-time wage workers, considering several related factors with weight; socio-demographic variables, health behavior-related variables, and occupational variables. Besides, we examined the risk of weight change caused by night work by dividing into weight loss and weight gain group and further analysis after stratification by working hours in male and female workers.

## Conclusions

In this cross-sectional study with national representative sample, night work may lower the risk of weight loss in male workers and induce weight change (weight loss or weight gain) in female workers with long working hours. Therefore, future studies with cohort study design for night workers are needed to reveal the mechanisms and health effects of weight change associated with night work through monitoring health risk behaviors including smoking, drinking, nutrition intake, physical activities, and consideration of other related occupational factors and establish proper management solutions with health and labor policies for Korean night workers.
